# Continuous glucose monitor metrics and hemoglobin A1c correlation in youth with diabetes: A retrospective analysis of real‐world correlations

**DOI:** 10.1111/1753-0407.13602

**Published:** 2024-09-12

**Authors:** Jessica A. Schmitt, Meryl C. Nath, Joshua Richman, Joycelyn Atchison

**Affiliations:** ^1^ Department of Pediatrics University of Alabama at Birmingham Heersink School of Medicine Birmingham Alabama USA; ^2^ University of Alabama at Birmingham Heersink School of Medicine Birmingham Alabama USA; ^3^ Department of Surgery University of Alabama at Birmingham Heersink School of Medicine Birmingham Alabama USA

Dear Editor,

Although factors other than glucose affect glycosylated hemoglobin A1c (HbA1c),^1^, ^2^, ^3^ HbA1c is used to monitor glycemia.^4^ Studies have shown racial differences^5^ and variations^6^ in continuous glucose monitor (CGM)‐measured mean glucose and laboratory HbA1c values. CGM use in youth has increased^7^; however, optimal utilization of CGM metrics as a proxy for HbA1c, particularly in specific populations, remains uncertain.

We aimed to evaluate the correlation of CGM metrics to HbA1c in youth with type 1 diabetes, identify the strongest correlation, and determine if patient characteristics significantly mitigate correlations. We reviewed data from non‐Hispanic White (NHW) and non‐Hispanic Black (NHB) youth with type 1 diabetes at Children's of Alabama from July 2019 to January 2022. Data included HbA1c, demographics, duration of diabetes, type of insulin administration, and CGM data from 14 and 90 days before HbA1c measurement. As the goal was to assess the correlation of CGM metrics and HbA1c in real‐world use, there was no requirement for days or percentage of days for CGM use for data inclusion.

Demographics and metrics were inspected by summary statistics and compared between groups using distribution‐appropriate bivariate tests. We examined smoothed scatterplots between each metric and HbA1c stratified by subject characteristics. After plots suggested no important nonlinear trends or interactions, we identified which metrics were most strongly related to HbA1c using linear regression and repeated this for subcohorts stratified by patient characteristics including: HbA1c cohorts (adequate, moderate, and poor glycemic management as defined by HbA1c: <7.5% [58 mmol/mol], 7.5%–9.5% [58–80 mmol/mol], and >9.5% [80 mmol/mol]), race, sex, age, and duration of diabetes. Finally, for high‐ranking measures, we fit regression models adjusted for CGM metric along with patient characteristics to check whether the model coefficient of the metric changed appreciably.

In total, 205 youth were included. Forty‐four (21.5%) were NHB, in line with the demographics of this clinic.^8^ A minority (*n* = 45, 22.0%), were publicly insured. Median age was 16.5 years (interquartile range [IQR]: 14.0–18.1) with a duration of diabetes of 5.7 years (IQR: 2.8–10.2). Ninety‐eight (47.8%) were female, and approximately half (*n* = 94, 49.7%) used an insulin pump. Eighty‐three (40.5%) were in the lowest HbA1c cohort, 42 (20.5%) were in the mid‐HbA1c cohort, and 80 (39.0%) were in the highest HbA1c cohort.

Except for coefficient of variation, all CGM metrics were strongly associated with HbA1c with 90‐day mean glucose being the most strongly correlated (*r* ^2^ = 0.79, *p* < 0.01), followed by 90‐day glucose management index (*r* ^2^ = 0.77, *p* < 0.01) (see Table 1). Analysis by HbA1c cohort showed variation in which metric most strongly correlated (see Table 1). Examination of smoothed scatterplots showed no indication that relationships between CGM metrics and HbA1c differed by race, sex, age and duration of diabetes.

**TABLE 1:** Adjusted *r* ^2^ of CGM metrics and HbA1c *n* = 205.

	Adjusted *r* ^2^	*p*‐Value for correlation	Other notes
**14‐day GMI** ***n* = 130**	**0.75**	**<0.01**	**Strongest correlation with HbA1c in those with an HbA1c <7.5% (58 mmol/mol)**
14‐day mean glucose *n* = 193	0.67	<0.01	
14‐day CV *n* = 193	0.01	0.06	
14‐day SD *n* = 193	0.45	<0.01	
14‐day time in range *n* = 193	0.64	<0.01	
14‐day time above 250 mg/dL (13.9 mmol/L) *n* = 193	0.65	<0.01	
**90**‐**day GMI** ***n* = 139**	**0.77**	**<0.01**	**Strongest correlation with HbA1c in those with an HbA1c >9.5% (80 mmol)**
**90**‐**day mean glucose** ***n* = 204**	**0.79**	**<0.01**	**Strongest correlation with HbA1c across total cohort**
90‐day CV *n* = 204	0.00	0.87	
90‐day SD *n* = 204	0.43	<0.01	
90‐day time in range *n* = 204	0.74	<0.01	
**90**‐**day time above 250 mg/dL (13.9 mmol/L)** ***n* = 204**	**0.76**	**<0.01**	**Strongest correlation with HbA1c in those with an HbA1c 7.5%–9.5% (58–80 mmol/mol)**

*Note*: Bolded text in column “Other Notes” highlights the strongest correlation by HbA1c cohort.

Abbreviations: CGM, continuous glucose monitors; CV, coefficient of variation; GMI, glucose management index; HbA1c, hemoglobin A1c; SD, standard deviation.

Ideally, both CGM and HbA1c are available. If not, our data suggest that, especially for those with a history of hyperglycemia, rather than defaulting to 14‐day GMI, the 90‐day GMI and mean glucose be used. As we continue to utilize CGM and remote monitoring, identifying which CGM metric best evaluates the individual patient's glycemia continues to be an area of interest.

## CONFLICT OF INTEREST STATEMENT

The authors declare no conflicts of interest.

## Data Availability

The data that support the findings of this study are available from the University of Alabama at Birmingham (UAB). Restrictions apply to the availability of these data, which were used under license for this study. Deidentified data are available from the authors with the permission of UAB.
